# A new species of *Rhodnius* from Brazil (Hemiptera, Reduviidae, Triatominae)

**DOI:** 10.3897/zookeys.675.12024

**Published:** 2017-05-18

**Authors:** João Aristeu da Rosa, Hernany Henrique Garcia Justino, Juliana Damieli Nascimento, Vagner José Mendonça, Claudia Solano Rocha, Danila Blanco de Carvalho, Rossana Falcone, Maria Tercília Vilela de Azeredo Oliveira, Kaio Cesar Chaboli Alevi, Jader de Oliveira

**Affiliations:** 1 Faculdade de Ciências Farmacêuticas, Universidade Estadual Paulista “Júlio de Mesquita Filho” (UNESP), Araraquara, SP, Brasil; 2 Departamento de Vigilância em Saúde, Prefeitura Municipal de Paulínia, SP,Brasil; 3 Instituto de Biologia, Universidade Estadual de Campinas (UNICAMP), Campinas, SP, Brasil; 4 Departamento de Parasitologia e Imunologia, Universidade Federal do Piauí (UFPI), Teresina, PI, Brasil; 5 Instituto de Biociências, Letras e Ciências Exatas, Universidade Estadual Paulista “Júlio de Mesquita Filho” (UNESP), São José do Rio Preto, SP, Brasil

**Keywords:** Brazil, cytotaxonomy, new species, *Rhodnius*, taxonomy, Triatominae

## Abstract

A colony was formed from eggs of a *Rhodnius* sp. female collected in Taquarussu, Mato Grosso do Sul, Brazil, and its specimens were used to describe *R. taquarussuensis* **sp. n.** This species is similar to *R. neglectus*, but distinct characters were observed on the head, thorax, abdomen, female external genitalia and male genitalia. Chromosomal differences between the two species were also established.

## Introduction

In the subfamily Triatominae, the genera *Panstrongylus* (15 species), *Triatoma* (74 species) and *Rhodnius* (20 species) are of particular epidemiological importance, although the other 15 genera (containing 43 species) can also transmit *Trypanosoma cruzi*, which is the etiological agent of Chagas disease (Poinar 2013, Galvão 2014, Mendonça et al. 2016, Souza et al. 2016). Among the 152 species within the subfamily there are two fossils: *T. dominicana* Poinar, 2005 and *P. hispaniolae* Poinar, 2013.

The first two species identified as belonging to the genus *Rhodnius* were described by Stal (1859): *R. nasutus* and *R. prolixus*. From that year until 1979, a total of 12 species were identified (Lent and Wygodzinsky 1979). In 2003, Galvão et al. considered 16 valid species. The 17^th^, 18^th^, 19^th^ and 20^th^ species in that genus were respectively *R. zeledoni* Jurberg et al. 2009; *R. montenegrensis* Rosa et al. 2012; *R. barretti* Abad-Franch et al. 2013, and *R. marabaensis* Souza et al. 2016.

Most *Rhodnius* species live in palm trees, and several cases of transmission of Chagas disease have been associated with the consumption of açaí containing feces of triatomines infected by *T. cruzi* (Ferreira, Branquinho & Leite, 2014; Ministério da Saúde, Brasil 2017). Apart from such cases, which occur more frequently in the northern region of the country, it is worth noting that *R. neglectus* was found in palm trees (species of *Roystonea*, *Syagrus* and *Acrocomia*] in the city of Araçatuba, São Paulo, in 2009, as well as in palm trees (*Livistona australis*) located in the central square of the city of Monte Alto, São Paulo, in February 2012 (Rodrigues et al. 2014, Carvalho et al. 2013, respectively).

Based on morphological, morphometric and cytogenetic characters, this paper describes *R. taquarussuensis* sp. n., which is similar to *R. neglectus*. The first collected specimen of *R. taquarussuensis* was a female that invaded a domicile and laid eight eggs. The colony formed from those eggs resulted in the specimens used in this description.

## Materials and methods

### Morphological identification and description

**Figure 1.**
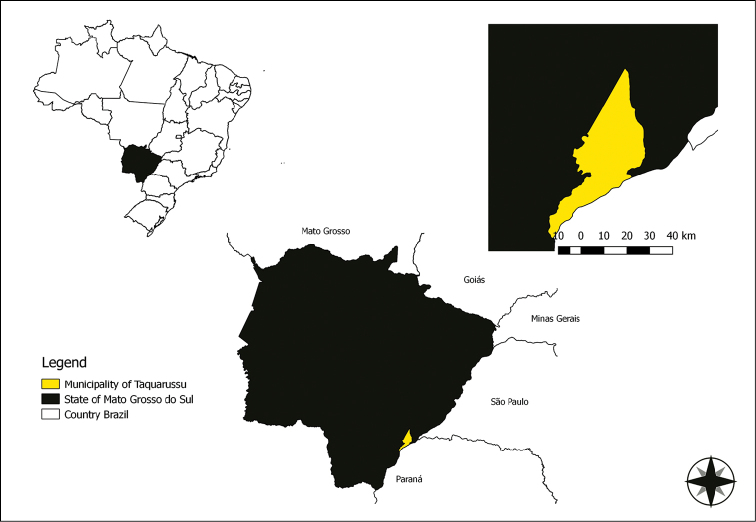
Localization of Taquarussu - MS where female of *R. taquarussuensis* sp. n. is collected (22°29'233"S, 053°21'107"W).

**Figure 2.**
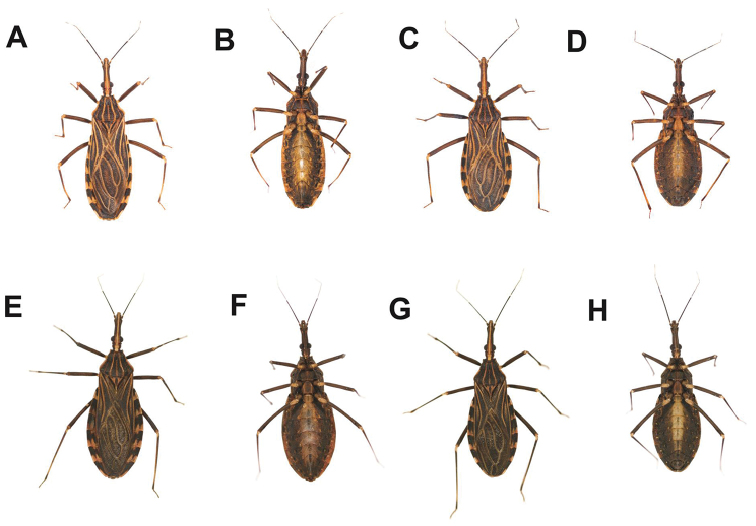
*R. taquarussuensis* sp. n. female **A** dorsal side **B** ventral side, *R. taquarussuensis* sp. n. male **C** dorsal side **D** ventral side, *R. neglectus* female **E** dorsal side **F** ventral side), *R. neglectus* male **G** dorsal side **H** ventral side.

On 10 November 2010 a female of *Rhodnius* sp. invaded a rural dwelling (22°29'07.7"S; 53°21'08.9'W) in the city of Taquarussu, Mato Grosso do Sul, Brazil, and was captured (Fig. 1). That specimen remained alive for a few days and laid eight eggs (Fig. 2). By means of macroscopic identification and subsequent optical microscopy (OM) and using the key of Lent and Wygodzinsky (1979), a clear similarity with *R. neglectus* was noticed. In view of that, all characters observed and documented for *Rhodnius* sp. were checked for *R. neglectus* CTA 229, which is a colony that has been kept since June 27, 2011 at the Triatominae Insectarium of the Faculty of Pharmaceutical Sciences, UNESP, Araraquara (FCFAR/UNESP). The temperature, humidity and light cycle conditions are not controlled due to the insect’s biodiversity, but these parameters are measured daily, varying the temperature between 20-35ºC and humidity 50-80%. Insects kept in colonies are fed directly on ducks every 15 days and consists of specimens from the Brazilian National and International Triatominae Taxonomy Reference Laboratory at the Oswaldo Cruz Institute in Rio de Janeiro, Brazil (LNIRTT). The colony was kindly provided by Dr. José Jurberg and Dr. Cleber Galvão, and the specimens that originated it were collected in northern Formoso, Goiás, Brazil.

A colony was formed from the eight eggs laid by the *R. taquarussuensis* sp. n. female and identified as Araraquara Triatominae Colony (CTA) 277. The specimens of that colony were used to describe *R. taquarussuensis* sp. n.

### Morphological study

**Figure 3.**
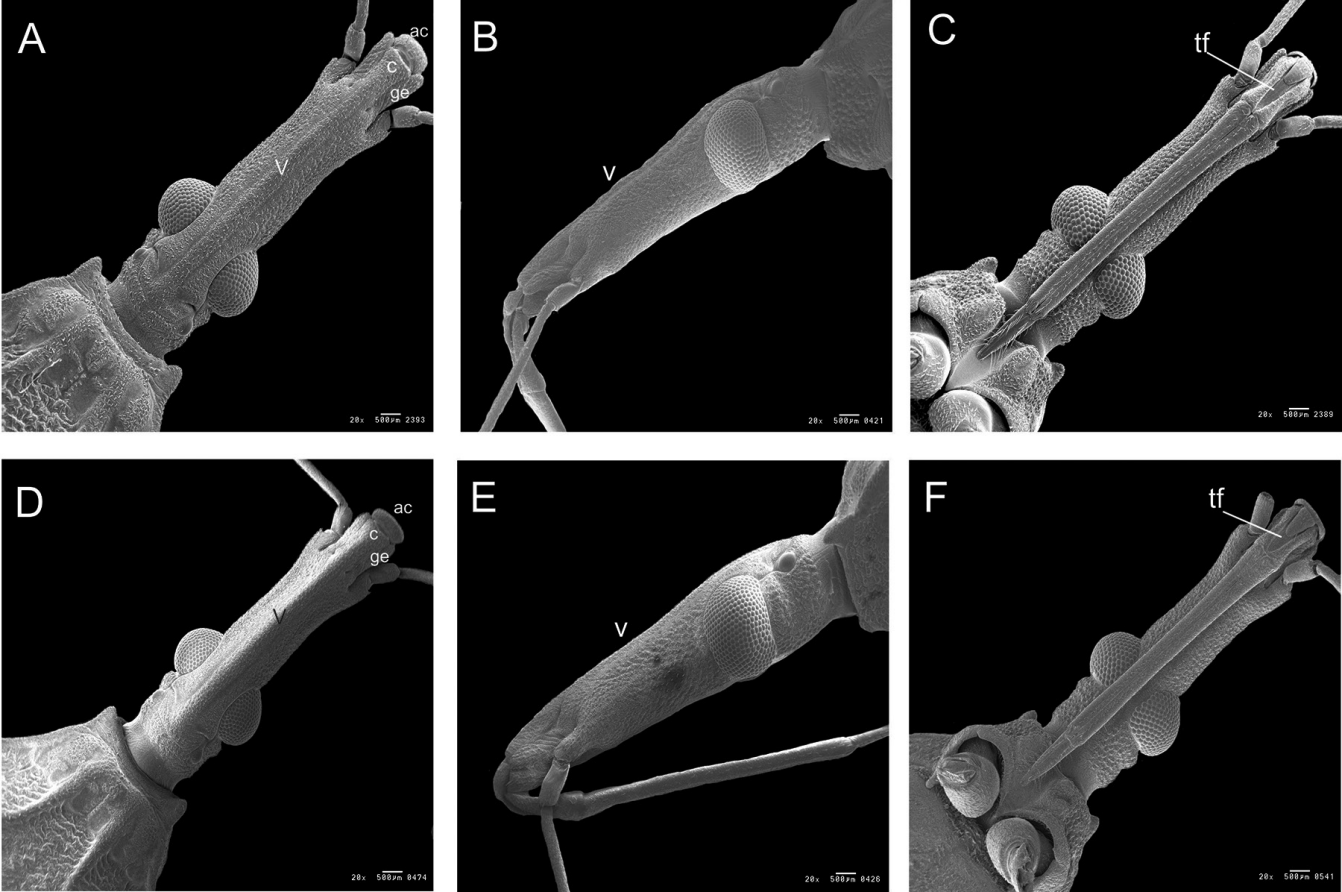
Head by SEM of *R. taquarussuensis* sp. n. **A** dorsal view **B** lateral view, **C** ventral view, *R. neglectus* **D** dorsal view **E** lateral view **F** ventral view. v: vertice, ge: gena, c: clypeus, ac: anteclypeus, tf: triangular furrow.

**Figure 4.**
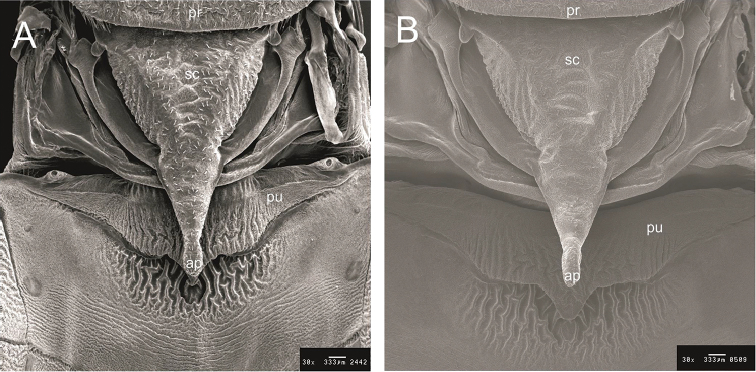
Escutellum and process of I urotergit by SEM. **A** *R. taquarussuensis* sp. n. **B** *R. neglectus*. pr: pronotum, sc: escutelum, pu: process of I urotergit, ap: apex of escutelum.

**Figure 5.**
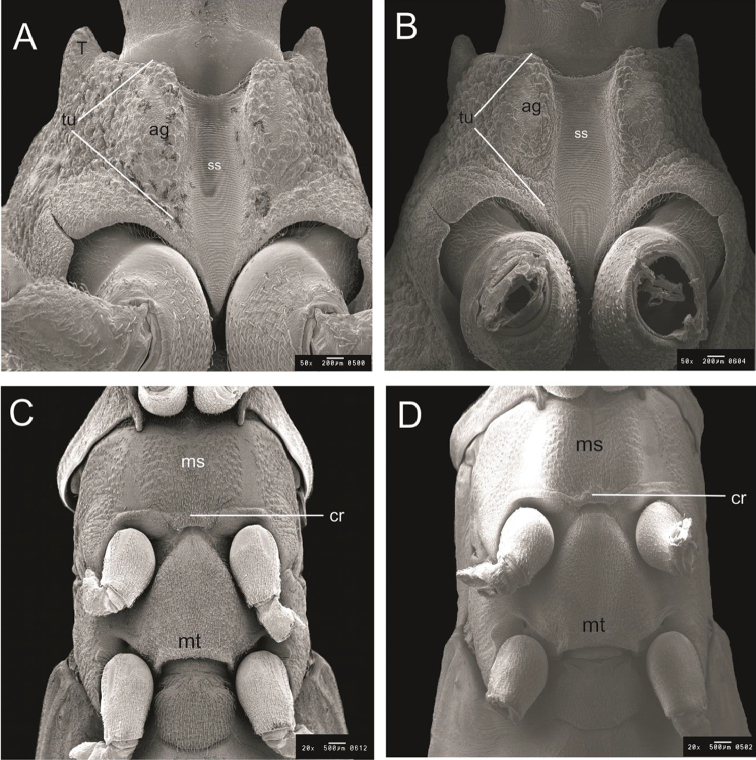
Thorax ventral by SEM. **A, C** *R. taquarussuensis* sp. n. **B, D** *R. neglectus*. ss: stridulatory sulcus, ms: mesosternum, mt: metasternum, tu: tubercle, ga: glabrous area, cr: central region.

**Figure 6.**
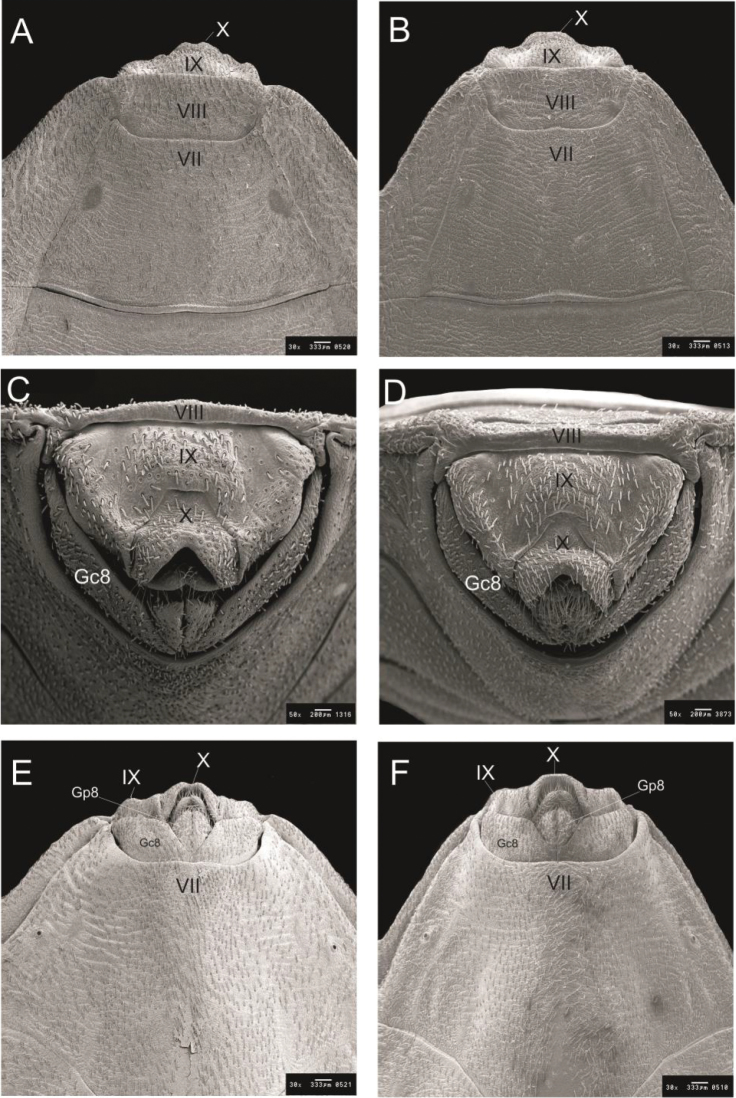
Female external genitalia by SEM *R. taquarussuensis* sp. n. **A** dorsal view, **C** posterior view **E** ventral view, *R. neglectus* **B** dorsal view **D** posterior view **F** ventral view. Gc 8: gonocoxite VIII; Gc 9: gonapophyse IX; Gp 8: gonapophyse VIII; VII, IX: tergites; X: segment.

**Figure 7.**
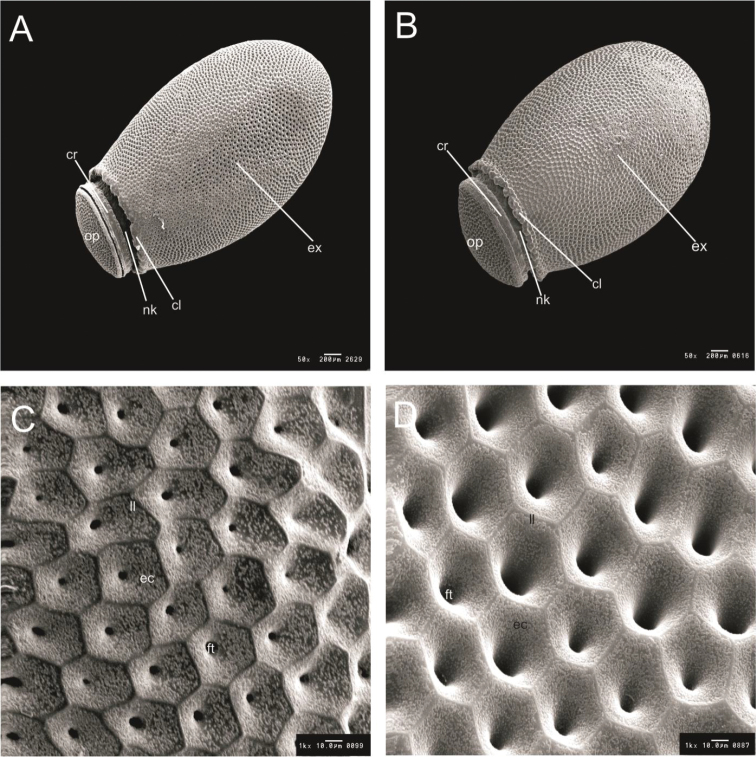
Eggs general vision and egg exochorium detail of *R. taquarussuensis* sp. n. (**A, C**), *R. neglectus* (**B, D**). cl: colar, cr: chorial rim, ex: exochorium, nk: neck, op: operculum, ec: exochorium cell, ft: follicular tubes, ll: limiting line.

**Figure 8.**
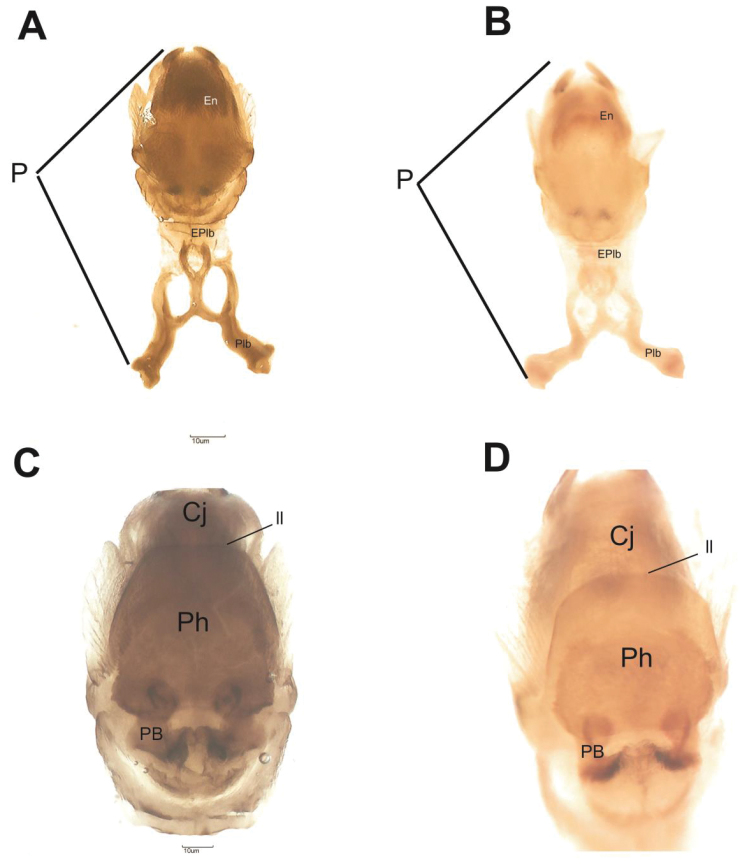
Phallus of *R. taquarussuensis* sp. n. **A** dorsal view **C** ventral view, *R. neglectus* **B** dorsal view **D** ventral view. Cj: conjunctive, En: endosome, EPlb: median extencion of basal plate, P: phallus, Plb: basal plate, PrG: gonopore process, PrPh: phallossoma process, Ph: phallosoma, PrCj: conjunctive process, ll: line limit.

**Figure 9.**
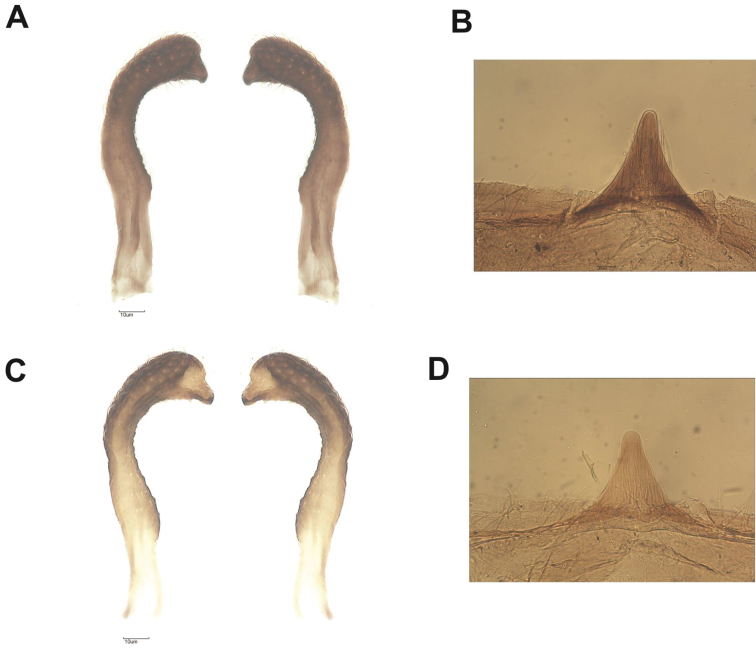
Parameres dorsal view of *R. taquarussuensis* sp. n. (**A**), Median process of the pygophore of *R. taquarussuensis* sp. n. (**B**) Parameres dorsal view of *R. neglectus* (**C**) Median process of the pygophore of *R. neglectus* (**D**).

The morphological study by OM and scanning electron microscopy (SEM) consisted of the observation of the head, thorax and abdomen of 30 adult females and 30 adult males, as well as 40 eggs of *R. taquarussuensis* sp. n. and the same number of specimens of *R. neglectus*, according to Barata (1981), Quintero (2003), Obara et al. (2007), Rosa et al. (2012), Rosa et al. (2014), Souza et al. (2016) (Figs 3–9).

Female external genitalia were observed from the dorsal, posterior, and ventral sides (Fig. 6) by SEM, according to Rosa et al. (2010). The study of the male genitalia was carried out by OM (Figs 8, 9), following a technique developed by Jader de Oliveira based on Gallati (2016). The denominations used were those defined by Lent and Jurberg (1969).

The Leica MZ APO stereoscope from the Faculty of Pharmaceutical Sciences, UNESP, Araraquara, and the scanning electron microscope Topcon SM-300 located in the Department of Physical Chemistry at the Chemistry Institute, UNESP, Araraquara, were used for observation and capture of images.

### Morphometric study

In the morphometric study by OM, 15 egg shells, 15 females and 15 males from the colony were measured, the same being done for *R. neglectus* CTA 229 (Table 1).The parameters measured were: total length, width of thorax and abdomen, length of the scutellum, three segments of the proboscis and four segments of the antenna, as well as five parameters of the head following Dujardin et al. (1999). Eggs had their length and the diameter of the opercular opening measured. The wings of *R. taquarussuensis* sp. n. and *R. neglectus* were studied by geometric morphometry using seven anatomical landmarks, according to parameters established by Gurgel-Gonçalves et al. (2008), as well as based on Rosa et al. (2012).

**Table 1.** Mean of measurement (mm) of 15 females and 15 males of *R. taquarussuensis* sp. n. and *R. neglectus*.

Female			Male	
	*R. taquarussuensis*	*R. neglectus*	*R. taquarussuensis*	*R. neglectus*
**TL**	17,25	17,25	**15,24**	**15,96**
**MLA**	9,86	10,03	**8,41**	**9,05**
**MLT**	**5,00**	**4,11**	**4,54**	**3,79**
**R1**	**0,75**	**0,92**	**0,71**	**0,92**
**R2**	**3,38**	**3,58**	**3,16**	**3,59**
**R3**	**0,85**	**0,95**	**0,77**	**0,96**
**HL**	**4.44**	**5,81**	**4.20**	**5,24**
**EO**	**1.53**	**1,98**	**1.42**	**1,80**
**IE**	**0.61**	**0,77**	**0.54**	**0,66**
**PO**	**1.00**	**3,67**	**1.04**	**3,37**
**AO**	**2.62**	**0,90**	**2.44**	**0,91**
**AT**	**1.93**	**2,30**	**1.82**	**2,30**
**SC**	1,96	2,03	1,69	1,86
**A1**	0,45	0,55	0,29	0,59
**A2**	3,56	4,13	2,24	4,35
**A3**	2,03	2,43	1,28	2,50
**A4**	1,36	1,82	0,87	1,92
**Eggs**	***R. taquarussuensis***		***R. neglectus***	
**TE**	**1,72**		**1,62**	
**OO**	**0,49**		**0,52**	

*30 eggshells were used for each species.

**TL**, Total length of the triatomine; **MLA**, maximum length of the abdomen; **MLT**, maximum length of the thorax; **R1**, **R2** and **R3**, lengths of first, second, and third rostral segments, respectively; **HL**, head length; **EO**, external distance between ocelli; **IE**, inner distance between eyes; **PO**, postocular distance (excluding neck); **AO**, anteocular distance; **AT**, antenniferous tubercle; **SC**, Scutellum; **A1**, **A2**, **A3** and **A4**, 1st, 2nd, 3rd, and 4th left antennal segments, respectively; **TE**, Total egg length; **OO**, egg opercular opening. The values in bold were significant at α = 0.05, using unpaired t-test.

The observations and measurements were carried out on a Leica MZ APO stereoscope and the Motic Images Advanced System version 3.2.

### Cytogenetic identification

In this study ten male specimens of *R. taquarussuensis* sp. n. were used for C and CMA_3_/DAPI–banding analyses and ten male specimens of *R. neglectus* were used for CMA_3_/DAPI–banding analyses. After being lacerated and placed on the slide, the seminiferous tubules underwent cytogenetic procedures following the C-banding (Sumner 1972) and CMA_3_/DAPI-banding protocols [Schimid 1980, with modifications according to Severi-Aguiar et al. 2006]. C-banding was analyzed under a Jenaval (Zeiss) MO connected to a digital camera and the Axio Vision LE 4.8 image analyzer (Copyright ©2006-2009 Carl Zeiss Imaging Solutions Gmb H), whereas CMA_3_/DAPI-banding was analyzed using Zeiss-Axioskop and Olympus BX-FLA fluorescence microscopes (FM).

## Taxonomy

### Family Reduviidae Latreille, 1807

#### Subfamily Triatominae Jeannel, 1919

##### Genus *Rhodnius* Stål, 1859

###### Rhodnius taquarussuensis sp. n.

Taxon classificationAnimaliaHemipteraReduviidae

http://zoobank.org/16C7EE86-3C36-4BA9-BDFC-E914CC4C2F80

Figure 2

####### Holotype.

**BRAZIL: Mato Grosso do Sul**: Taquarussu; Residence, 22°29'07.7"S; 53°21'08.9'W, 10 November 2010 H. E. G. Justino. UNESP (♀).

####### Paratypes.

**BRAZIL**: Colony formed from eggs obtained from the holotype: Araraquara: Triatominae Insectarium of the Faculty of Pharmaceutical Sciences, Araraquara, January 3, 2017, J. A. da Rosa, UNESP (25 ♂ 25 ♀).

####### Additional paratypes.

CTIOC - Collection of Triatomines of the Oswaldo Cruz Institute, Rio de Janeiro - Brazil (2 ♂ 2 ♀). Entomological Reference Collection of the Faculty of Public Health - USP, São Paulo -Brazil (1 ♂ 1 ♀). Collection of the Institute of Entomology of the Metropolitan University of Education Sciences (IEUMCE), Santiago - Chile (2 ♂ 2 ♀).

####### Etymology.

The name *Rhodnius taquarussuensis* sp. n. was chosen because this species was found in the city of Taquarussu, Mato Grosso do Sul, Brazil.

####### Diagnosis.

*Rhodnius taquarussuensis* sp. n. is close to *R. neglectus*, their differences being the color and a variety of morphological, morphometric and cytogenetic characters (Tables 1, 2). The general color of *R. taquarussuensis* sp. n. is brown, whereas *R. neglectus* is dark brown, almost black. This difference is particularly noticeable on the hind wings. The stridulatory sulcus of *R. taquarussuensis* sp. n. is brown at the base and black on the sides, whereas on *R. neglectus* it is completely black.

**Table 2.** Main distinguishing characters between *R. taquarussuensis* sp. n. and *R. neglectus*.

Distinguishing characters		Species	
		*R. taquarussuensis*	*R. neglectus*
Overall color		Brown	Dark brown
Genae		Lengthier longer	Longer
Vertex		Quite visible	Not visible
Ventral triangular furrow		Filamentous way	Rounded way
Scutellum		Covers the final portion of the urotergite I process	The apex of the process of the urotergite I is perfectly visible
Stridulatory sulcus		Straight	Waisted
Mesothorax		Half-moon shaped and regular	Pronounced and slightly irregular
Female external genitalia	Dorsal side	10^th^ segment presents a concavity in the middle portion	10^th^ segment is straight
	Posterior side	The limits of the 9^th^ segment with gonocoxite VIII are curve	The limits of the 9^th^ segment with gonocoxite VIII are straight
	Ventral side	There is a concavity in the external limit with the 10^th^ segment	There is a straight line in the external limit with the 10^th^ segment
Male genitalia	Phallothecal sclerite	Trapezoidal shape	Rounded shape
	Tip of parameres	Thinner	Thin
Heterochromatin in the autosomes		Present	Absent
CMA^+^ in autosomes		Present	Absent

On the head, differences were noticed on the vertex, genae, antennae and triangular furrow of the first segment of the rostrum. The vertex of the head of *R. taquarussuensis* sp. n. is quite visible, whereas on *R. neglectus* it is not (Fig. 3A, B, D, E). The genae of *R. taquarussuensis* sp. n. are longer than those of *R. neglectus* (Fig. 3A, D).On *R. taquarussuensis* sp. n. the 10^th^ part of the second segment of the antenna is brown; on *R. neglectus*, though, only the basis has that color. The triangular furrow of the first segment of the rostrum, towards the second segment, ends in a filamentous way on *R. taquarussuensis* sp. n. and in a rounded way on *R. neglectus* (Fig. 3C, F). On the thorax, differences can be found on the pronotum, wings, scutellum, prosternum, mesosternum and metasternum (Figs 4, 5). The membranous portion of the hind wings is brown on *R. taquarussuensis* sp. n. and dark brown on *R. neglectus*. The scutellum ends in a rounded apex on *R. taquarussuensis* sp. n. and in a filamentous apex on *R. neglectus* (Fig. 4A, B). On *R. taquarussuensis* sp. n. the apex of the scutellum covers the final portion of the urotergite I process, while on *R. neglectus* the apex of the process of the urotergite I is perfectly visible (Fig. 4A, B). The lines limiting the stridulatory sulcus are straight on *R. taquarussuensis* sp. n. and narrowed in the anterior third on *R. neglectus* (Fig. 5A, B). On *R. taquarussuensis* sp. n. the basis of the stridulatory sulcus is brown and the sides are black, whereas on *R. neglectus* the entire stridulatory sulcus is black. The central region of the limit between the mesosternum and the metasternum is regular and half-moon shaped on *R. taquarussuensis* sp. n., while on *R. neglectus* it is pronounced and slightly irregular (Fig. 5C, D). The beginning of the metasternum is narrow on *R. taquarussuensis* sp. n. and wide on *R. neglectus* (Fig. 5C, D). The ventral abdomen of *R. taquarussuensis* sp. n. is light brown, and that of *R. neglectus* is dark brown (Fig. 2). The terminal portion of the paramere of the male genitalia of *R. taquarussuensis* sp. n. is thinner than that of *R. neglectus* (Fig. 9A, C). The dorsal phallothecal sclerite has a trapezoidal shape on *R. taquarussuensis* sp. n. and is rounded on *R. neglectus* (Fig. 8C, D). The external limit of the 10^th^ segment of the dorsal side of the female external genitalia of *R. taquarussuensis* sp. n. presents a concavity in the middle portion, whereas on *R. neglectus* that limit is straight (Fig. 6A, B). From posterior view, the limits of the 9^th^ segment with gonocoxite VIII are curve on *R. taquarussuensis* sp. n. and straight on *R. neglectus*, and the superior line limiting the 10^th^ and 9^th^ segments is straight on *R. taquarussuensis* sp. n. and curve on *R. neglectus* (Fig. 6C, D). In the ventral side of the female external genitalia of *R. taquarussuensis* sp. n. there is a concavity in the external limit with the 10^th^ segment that is also noticed from dorsal view; on *R. neglectus* that limit is a straight line. From ventral view, the external limits of the 9^th^ segment of the female external genitalia are curve on *R. taquarussuensis* sp. n. and straight on *R. neglectus* (Fig. 6E, F).

Among the 19 characters measured, 12 showed significant differences between *R. taquarussuensis* sp. n. and *R. neglectus* in both sexes and also the eggs of both species. Two characters showed differences only between males, and five characters did not show significant differences (Tables 1, 2).

####### Description.

A total of 15 adult females and 15 adult males of *R. taquarussuensis* sp. n. and *R. neglectus* were measured, as well as 30 eggs shells of both species. Such measurements are detailed in Table 1.

The head of *R. taquarussuensis* sp. n. has a prominent brown vertex contrasting with the black sides. The clypeus is well defined. The genae are large, visible and dark brown, moving towards the anteclypeus (Figs 2A, C, 3 A, B). The limits between the genae and the clypeus are brown.

The first segment of the antennae is black with mixes of brown. The articulation between the first and second segment of the antennae is brown. Roughly all the 10^th^ part of the beginning of the second antennal segment is brown. The second segment is mostly black. In the articulation between the second and third antennal segment there is a black ring followed by a brown one. The beginning of the third segment (around 1/3) is black and the remaining portions (2/3) are brown. The articulation between the third and fourth antennal segment is brown. The beginning of the fourth segment is black and the remaining portions are brown with mixes of black (Fig. 2A, C).

The eyes are black and the ocelli are brown. The neck has a brown central dorsal strip flanked by two (1+1) black, narrower strips. The ventral portion of the neck between the ocelli is dark brown (Fig. 2A, B, C, D).

The pronotum of the thorax of *R. taquarussuensis* sp. n. has a trapezoidal shape and is limited by a brown carina. In the antero posterior direction the pronotum has other two brown carina in the middle portion and six black strips. The three carina and the three brown strips are interspersed with the six black strips, which are larger. The collar (first portion of the pronotum) in the central part is brown and is followed by two (1+1) black glabrous areas and the two (1+1) antero lateral angles. The anterior portion of the pronotum consists of three anterior lobes which are clearly distinct from the posterior portion (hindlobe). Those three anterior lobes are limited by the carina and on each of them there are two black glabrous areas with a lengthy and irregular outline (Fig. 2A, C).

The cuticle involving the veins of the hemelytron is light brown. The corium between the veins of the coriaceous region is dark brown, whereas that of the membrane is brown (Fig. 2A, C).

The prosternum contains the stridulatory sulcus, which moves along that segment in an antero-posterior direction, having a brown color in the background and black on the sides. Two elongated tubercles limit the anterior half of the stridulatory sulcus. In the superior portion and in diagonal direction from the tubercles there are two black glabrous areas surrounded by a set of brown sensilla (Fig. 5A).

The mesosternum is limited anteriorly by the prosternum and posteriorly by the metasternum, both limits being brown. The central line dividing two dark brown elevations is also brown. Those two elevations are limited by two (1+ 1) black side glabrous areas diagonally placed. The central region of the posterior limit of the mesosternum has a half-moon shape. The metasternum is brown and resembles an isosceles triangle. Its anterior portion, i.e., its limit with the mesosternum, corresponds to the vertex of the triangle and is narrow, whereas its posterior portion, i.e., its limit with the first abdominal segment, corresponds to the basis of the triangle (Fig. 5C).

The three pairs of coxae are brown, except for the black glabrous areas. The trochanters of the anterior pair of legs are brown, but mixed with black glabrous areas. The middle and posterior pairs of trochanters are brown and have no glabrous areas. The three pairs of femora are black and the same color prevails in the three pairs of tibiae, except in the articulations with the femur and the spongy fossula, which are brown. The spongy fossulae are located in the first and second pairs of legs in the final portion of the tibia, alongside the articulations with the tarsi (Fig. 2A, B, C, D).

The abdomen of *R. taquarussuensis* sp. n. presents a brown color in the longitudinal central portion. On the sides of each segment there are (3+3) black glabrous areas, which are mixed with brown and black areas. The connexivum of the dorsal portion lies between the second and seventh segment. For each of those segments the anterior half is black and the posterior one is brown. The dorsal connexivum, also lying between the second and seventh segment, has a black color in 2/3 of the anterior portion, but that black color ends in an irregular way over the remaining 1/3, which is brown. Therefore, the black portion of the connexivum presents two edges moving towards the brown portion: one in the internal limit of the connexivum and the other in the middle portion. However, the connexivum of the second dorsal segment is black in the anterior half and brown in the posterior one, the limit between the portions having a diagonal shape. The seventh segment, on the other hand, is practically all black, except for a small brown strip located in the external posterior half. Type 1 sensilla, which prevail on the head, thorax and abdomen, have a brown color (Fig. 2B, D).

Male genitalia have the typical aspect of the genus *Rhodnius*. The median process of the pygophore (PrP) is short and triangular, but the base is broad and the sides are elongated with a thin edge. Parameres are hairy with a thin edge. From ventral view, the phallosome (Ph) has a broad plate whose superior region has a trapezoidal shape and occupies the middle region of the aedeagus. The support of the phallosome plate (PrPh) is broad. Conjunctival process I (PrcjI) is present and II (PrcjII) is absent. Endosomal process (En) is well-developed when seen from dorsal and ventral view (Figs 8A, C, 9B).

The dorsal side of the female external genitalia presents a concavity in the middle portion of the 10^th^ segment. Seen from posterior view, the limits (1+1) of the 9^th^ segment with gonocoxite VIII are curve, whereas the superior line limiting the 10^th^and 9^th^segments is straight. In the central portion of the 10^th^ segment of the ventral side of the female external genitalia there is another concavity that can be noticed from dorsal view. The external limits (1+1) of the 9^th^ segment of the female external genitalia are curve when seen from ventral view (Fig. 6A, C, E).

Egg shells of *R. taquarussuensis* sp. n. have a length of 1.72 mm and an opercular opening of 0.49 mm. They present lateral flattening, collar and exochorion cells, most with pentagonal or hexagonal shape (Fig. 7A, C).

Finally, although *R. taquarussuensis* sp. n. showed the same number of chromosomes as *R. neglectus* and all the tribe Rhodniini, i.e., 2n = 22 (Figure 11B), the constitutive heterochromatin pattern and the composition of the pairs of bases of DNA rich in AT and CG were completely different from *R. neglectus*, as the analysis of the nuclei of the initial prophases of *R. taquarussuensis* sp. n. has revealed a chromocenter consisting of sex chromosomes (arrow) and several heterochromatic blocks dispersed in the nucleus (Fig. 11A). The analysis of metaphase I of *R. taquarussuensis* sp. n. has demonstrated that this triatomine has heterochromatic blocks in both extremities of practically all the autosomes and in the Y sex chromosome (Fig. 11B), unlike what has been recently stated for many populations of *R. neglectus* that do not present heterochromatin in autosomes (Alevi et al. 2015a). Furthermore, *R. taquarussuensis* sp. n. has the X sex chromosome rich in CG (Fig. 12C), the Y rich in AT (Fig. 12D) and various blocks rich in CG dispersed in the prophase nucleus (Fig. 12C), while *R. neglectus* only has the X sex chromosome rich in CG (Fig. 12A) and the Y rich in AT (Fig. 12B), which proves the genetic differences between the two *Rhodnius* species.

**Figure 10.**
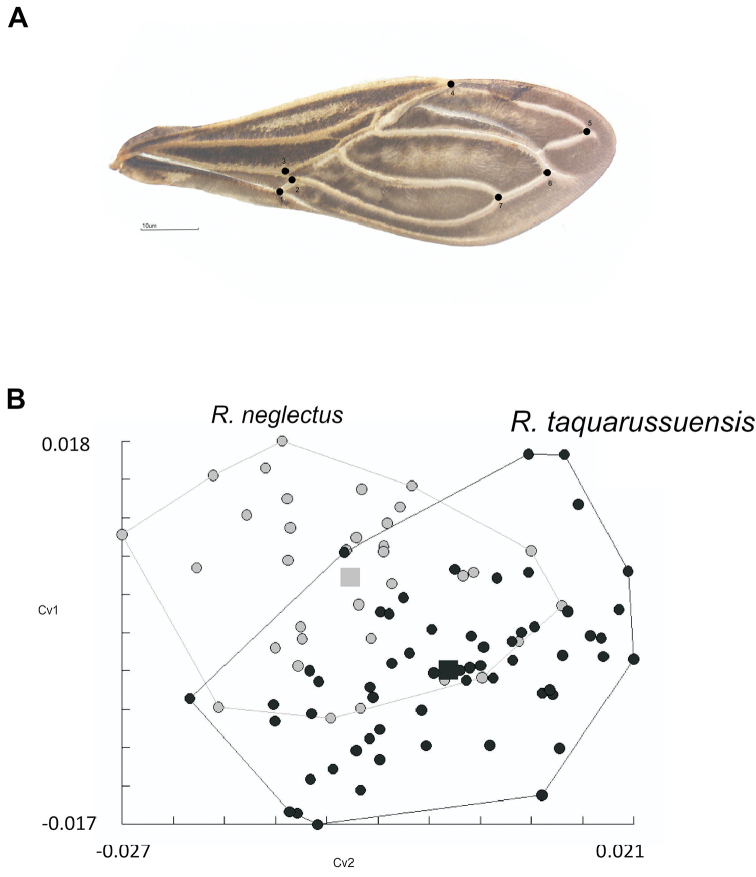
**A** Right wing of *R. taquarussuensis* sp. n. with the seven landmarks used in morphometric analysis. Following Bookstein (1990), all points correspond to type I landmarks (venation intersections) **B** Factorial maps in the plane of the two discriminant factors of wing shape variation (canonical variables 1 and 2, or CV1 and CV2) presenting the distribution of specimens of *R. taquarussuensis* sp. n. (Rta, black cicle) and *R. neglectus* (Rne, silver cicle).

**Figure 11.**
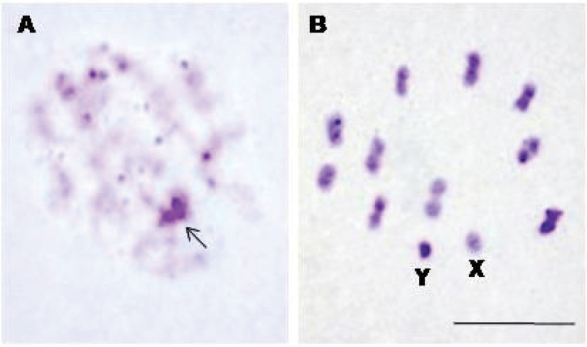
Constitutive heterochromatin pattern in *R. taquarussuensis*. **A** Initial prophases with a chromocenter heterochromatic consisting of sex chromosomes (arrow) and several heterochromatic blocks dispersed in the nucleus **B** Metaphase I with heterochromatic blocks in both extremities of practically all the autosomes and in the Y sex chromosome. X: X sex chromosome, Y: Y sex chromosome. Bar: 10 μm.

**Figure 12.**
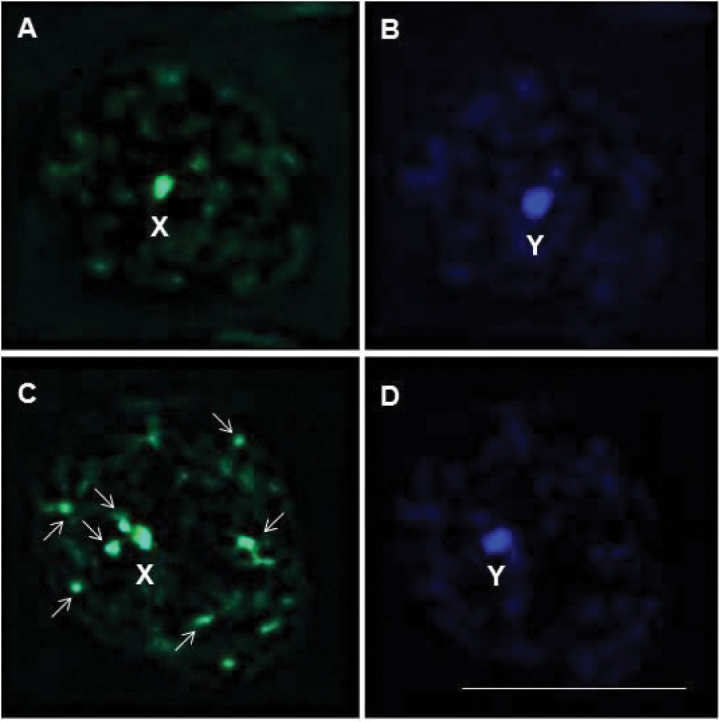
Composition of the pairs of bases of DNA rich in AT and CG in *R. neglectus* (**A, B**) and *R. taquarussuensis* (**C, D**). **A** X sex chromosome rich in CG **B** Y sex chromosome rich in AT **C** X sex chromosome and various blocks dispersed in the prophase nucleus (arrows) rich in CG **D** Y sex chromosome rich in AT. X: X sex chromosome, Y: Y sex chromosome. Bar: 10μm.

## Discussion

The subfamily Triatominae include 18 genera comprising 152 species, 20 of which belong to the genus *Rhodnius* (Galvão 2014, Mendonça et al. 2016, Souza et al. 2016).The difficulties involved in the specific identification of *Rhodnius* have already been noted by Neiva and Pinto (1923), as well as by Rosa et al. (2012) and Souza et al. (2016). However, even though it is difficult to specify the distinctions among the species of that genus, in the last eight years four species were described: *R. zeledoni*, *R. montenegrensis*, *R. barretti*, and *R. marabaensis*. Therefore, *R. taquarussuensis* sp. n. is the 21^st^ species of the genus and the 5^th^ described in the last eight years. Its similarity with *R. neglectus* was noticed after the capture of the first female specimen, as the most evident macroscopic characters, such as size, general aspect and connexivum, showed no differences. As a result, it was decided to base its description on the differences from *R. neglectus*.

In addition to the macroscopic characters, *R. taquarussuensis* sp. n. and *R. neglectus* were considered “close to” because the OM study indicated similar characters between them, including: placement of black and brown spots on the dorsal and ventral connexivum, length of the four segments of the antenna, pronotum, antero lateral angles, urotergite I process, geometric morphometry of the hind wings, median process of the pygophore and morphological characters of the eggs.

*Rhodnius taquarussuensis* sp. n. was considered distinct from *R. neglectus* on account of the observation of color, eleven morphological characters, twelve morphometric characters and cytogenetic features (Tables 1, 2). All those differences are consistent with Lent and Wygodzinsky (1979), whose descriptive key to 11 species of *Rhodnius* lists 13 morphological characters for specific distinction: color, tibia, legs, pronotum, head, posterior lobe of the pronotum, anterolateral angles, median process of the pygophore, connexivum, scutellum, eyes and antennae.

Regarding the color, the distinction between *R. taquarussuensis* sp. n. and *R. neglectus* was based on the general aspect, segments of the antenna, hind wings and stridulatory sulcus. The general color of *R. taquarussuensis* sp. n. is brown, whereas *R. neglectus* is dark brown, almost black, or “brown dark”, as referred to by Lent and Wygodzinsky (1979).

Out of the eleven morphological characters that distinguish *R. taquarussuensis* sp. n. from *R. neglectus*, three are located on the head: dorsal vertex, genae and triangular furrow of the first segment of the proboscis. The difference related to the vertex was one of the characters used by Souza et al. (2016) to distinguish *R. marabaensis* from *R. prolixus* and *R. robustus*. The differences between the genae for specific characterization are being reported for the first time in this description. The ventral triangular furrow was mentioned by Rosa et al. (1999) in their study of *T. rubrovaria* and it was mapped by Lent and Wygodzinsky (1979), but it was not named and it is being used for the first time as a distinctive character.

In what refers to the thorax, differences on the scutellum, protothorax, mesothorax, and metathorax were noticed. The scutellum of *R. taquarussuensis* sp. n. and *R. neglectus* differs in the shape of the apex and also the position on which that apex reaches urotergite I, since there is no significant difference in terms of length (Table 1). The taxonomic importance of the scutellum was tackled by Obara et al. (2007), who verified the differences of that character in eight species of *Triatoma*. Rosa et al. (2012) and Souza et al. (2016) also used it to describe *R. montenegrensis* and *R. marabaensis*, respectively.

The differentiation of seven genera of triatomines based on the shape of the prosternal stridulatory sulcus was carried out by Lent and Wygodzinsky (1979) and also by Souza et al. (2016) in the description of *R. marabaensis*. The description presented herein points out color and morphological differences between *R. taquarussuensis* sp. n. and *R. neglectus*. The mesothorax and metathorax, which have been used by Souza et al. (2016) to describe a new species, were found to be different in *R. taquarussuensis* sp. n. and *R. neglectus*.

Lent and Jurberg (1969) observed specific features in the characters of the male genitalia of 10 species of *Rhodnius* and since then that structure has been used to describe species of other genera of Triatominae, e.g., *Mepraia parapatrica* Frias, 2010, *P. mitarakaensis* Bérenger & Blanchet, 2007 and *T. jatai* Gonçalves et al., 2013. In the case of *R. taquarussuensis* sp. n. and *R. neglectus*, the difference in the male genitalia is the shape of the phallosome. With respect to the female external genitalia, differences between *R. taquarussuensis* sp. n. and *R. neglectus* could be observed on the dorsal, posterior and ventral sides, which differ from other 13 *Rhodnius* species, according to Rosa et al. (2014).

The eggs of *R. taquarussuensis* sp. n. and *R. neglectus* showed differences in the measurement of their length and opercular opening, the same as *R. montenegrensis* and *R. marabaensis* on the occasion of their description. As for the morphological characters, no differences were recorded. On the other hand, it should be noted that morphological differences were found by Barata (1981) in eggs of 10 *Rhodnius* species, by Santos et al. (2009) in three species, by Rosa et al. (2012) in the description of *R. montenegrensis* and by Santos et al. (2016) when describing *R. marabaensis*.

Dujardin et al. (1999) established the geometric morphometry of the hind wings as a distinctive character among Triatominae while studying the sexual dimorphism of *R. domesticus* and *T. infestans*. The technique proved valid, for instance, to indicate the distinction between *Mepraia spinolai* and *M. gajardoi*; *T. bahiensis* and *T. lenti*; *R. colombiensis*, *R. ecuadoriensis* and *R. pallescens*; five populations of *T. patagonica* (Campos et al. 2011, Díaz et al. 2014, Nattero et al. 2016). However, even though that technique has contributed to distinguish even very close species, it showed no significant results to distinguish *R. taquarussuensis* sp. n. from *R. neglectus*.

According to Justi and Galvão (2016) the group *R. prolixus* comprise the following species: *R. barretti*, *R. dalessandroi*, *R. domesticus*, *R. marabaensis*, *R. milesi*, *R. montenegrensis*, *R. nasutus*, *R. neglectus*, *R. neivai*, *R. prolixus* and *R. robustus*. Since *R. taquarussuensis* sp. n. is close to *R. neglectus* we suggest the inclusion of *R. taquarussuensis* sp. n. in the *R. prolixus* group and we present the main differences between the twelve species (Table 3).

**Table 3.** Distinguishing characters among twelve species of the group *Rhodnius prolixus*.

Species	Distinctive characters	References
*R. barretti*	The third antennal segment appears to be relatively shorter. The scutellar process is narrowly pointed.	Abad-Franch et al. 2013
*R. dalessandroi*	Antenniferous tubercle slightly pilose and with triangular glabrous depression in the upper region. Semicircular spot on the posterior end of the neck.	Carcavallo and Barreto 1976
*R. domesticus*	Head comparatively long, distinctly longer that pronotum. Process of pygophore rectangular.	Lent and Wygodzinsky 1979
*R. marabaensis*	The second antennal segment is 10.3 times larger than the first. The scutellum is larger and includes two prominent internal lateral carinea.	Souza et al. 2016
*R. milesi*	The male genitalia presents a second process of the phallosoma. Divergent antennal tubercle with an apical denticle.	Valente et al. 2001
*R. montenegrensis*	Anterior wings with well-demarcated veins, notable the Sc by a yellow tonality. Abdomen presents yellow spots interposed with dark ones over the ventral abdomen lengthwise.	Rosa et al. 2012
*R. nasutus*	Overall color light reddish brown, trochantera not contrasting conspicuously with femora. Median process of pygophore wide at base.	Lent and Wygodzinsky 1979
*R. neglectus*	Overall color dark brown, trochantera very light colored. Median process of pygophore narrow at base.	Lent and Wygodzinsky 1979
*R. neivai*	Pronotum entirely dark brown or black, including the carine. Connexivum blackish, with very small reddish spots.	Lent and Wygodzinsky 1979
*R. prolixus*	Anteocular region slightly over three times as long as postocular. Distance between eyes dorsally larger than width of eyes in dorsal view.	Lent and Wygodzinsky 1979
*R. robustus*	Anteocular region about four times as long as postocular. Specimens distance between eyes dorsally smaller than, or equal to, width of eye in dorsal view.	Lent and Wygodzinsky 1979
*R. taquarussuensis* sp. n.	Head with a prominent brown vertex contrasting with the black sides. The phallosome (Ph) has a broad plate whose superior region has a trapezoidal shape and occupies the middle region of the aedeagus.	This work

*group *R. prolixus* according to Justi and Galvão 2016.

Cytogenetic analyses of *R. taquarussuensis* sp. n. made it possible to describe the karyotype (2n = 22) and observe the constitutive heterochromatin pattern in the chromosomes (extremities of most autosomes), which are rich in CG. All the species in the tribe Rhodniini have 22 chromosomes (Alevi et al. 2013, 2015b). On the other hand, out of the 14 species of the genus *Rhodnius* whose chromosomes have been studied in the literature, only four show heterochromatic blocks in the autosomes, namely *R. colombiensis*, *R. nasutus*, *R. pallescens* and *R. pictipes* (Dujardin et al. 2002). *R. neglectus*, which is a similar species for *R. taquarussuensis* sp. n., does not show heterochromatic blocks in the autosomes (Dujardin et al. 2002; Panzera et al. 2012; Alevi et al. 2015a).

Although the evolutionary process in triatomine is disruptive (Dujardin et al. 2009) and intraspecific chromosome variation has been described for *R. ecuadoriensis* (Pita et al. 2013), *R. pallescens* (Gómez-Palacio et al. 2008), *P. geniculatus* (Crossa-Pérez et al. 2002), *T. dimidiata* (Panzera et al. 2006) *T. infestans* (Panzera et al. 2004, 2012) and *T. sordida* (Panzera et al. 1997), generally the distribution of species is associated with different countries [for exemple, *R. ecuadoriensis* from Peru and Ecuador (Pita et al. 2013) and *T. sordida* from Brazil and Argentina (Panzera et al. 1997)] or different regions [for example, *R. pallescens* from North and West regions from Colombia (Gómez-Palacio et al. 2008) and *T. infestans* from Andean group and Non-Andean group (Panzera et al. 2004, 2012)]. However, a population study was previously performed with *R. neglectus* (endemic species of Brazil) coming from different Brazilian states (Alevi et al. 2015a) and the authors pointed out that there is no intraspecific chromosome variation for this species. This fact and the morphological data described sustain the specific status of *R. taquarussuensis* sp. n., since the gain and loss of heterochromatin in the autosomes of *Rhodnius* are adaptive processes that can be linked to speciation processes, as recently noted for the group *pallescens* (Alevi et al. 2015c).

## Authors’ contributions

Conceived the study: JAR, HHGJ, JO and KCCA.Colected the bugs:HHGJ.

Prepared samples: JAR, JDN, JO, DBC and JDN. Analysed data: JAR, JO,VJM, RF,CSR and MTAO. Interpreted data: JAR, JO, KCCA, DBC. Wrote the manuscript: JAR, JO and KCCA.

All authors read and approved the final version of the manuscript.

## Supplementary Material

XML Treatment for Rhodnius taquarussuensis

## References

[B1] Abad-Franch F Pavan MG Jaramillo O Palomeque FS Dale C Chaverra D Monteiro FA (2013) *Rhodnius barretti*, a new species of Triatominae (Hemiptera: Reduviidae) from western Amazonia. Memórias Instituto Oswaldo Cruz 108: 92–99. http://dx.doi.org/10.1590/0074-0276130434 10.1590/0074-0276130434 PMC4109185 24473808

[B2] Alevi KCC Rodas LAC Tartarotti E Azeredo-Oliveira MTV Guirado MM (2015a) Entoepidemiology of Chagas disease in the Western region of the State of São Paulo from 2004 to 2008, and cytogenetic analysis in *Rhodnius neglectus* (Hemiptera, Triatominae). Genetics and Molecular Research 14(2): 5775–5784. http://dx.doi.org/10.4238/2015.May.29.9 26125776 10.4238/2015.May.29.9

[B3] Alevi KCC Ravazi A Mendonça VJ Rosa JA Azeredo-Oliveira MTV (2015b) Karyotype of *Rhodnius montenegrensis* (Hemiptera, Triatominae). Genetics and Molecular Research 12: 222–226. http://dx.doi.org/10.4238/2015.January.16.5 10.4238/2015.January.16.5 25729953

[B4] Alevi KCC Ravazi A Franco-Bernardes MF Rosa JA Azeredo-Oliveira MTV (2015c) Chromosomal evolution in the *pallescens* group (Hemiptera, Triatominae). Genetics and Molecular Research 14: 12654–12659. http://dx.doi.org/10.4238/2015.October.19.9 26505416 10.4238/2015.October.19.9

[B5] Alevi KCC Rosa JA Oliveira MTVA (2013) Mini Review: Karyotypic Survey in Triatominae Subfamily (Hemiptera, Heteroptera). Entomology, Ornithology & Herpetology: Current Research 2: 2 http://dx.doi.org/10.4172/2161-0983.1000106

[B6] Barata JM (1981) Aspectos morfológicos de ovos de triatominae: II – Características macroscópicas e exocoriais de dez espécies do gênero *Rhodnius* Stal, 1859 (Hemiptera – Reduviidae). Revista Saúde Pública 15: 490–542. https://doi.org/10.1590/S0034-89101981000500006 10.1590/s0034-89101981000500006 7048506

[B7] Bérenger JM Blanchet D (2007) A new species of the genus *Panstrongylus* from French Guiana (Heteroptera; Reduviidae; Triatominae). Memórias do Instituto Oswaldo Cruz 102: 733–736. http://dx.doi.org/10.1590/S0074-02762009000800007 17924003 10.1590/s0074-02762007005000088

[B8] Campos R Botto-Mahan C Coronado X Jaramillo N Panzera F Solari A (2011) Wing shape differentiation of *Mepraia* species (Hemiptera: Reduviidae). Infection, Genetics and Evolution 11: 329–333. https://doi.org/10.1016/j.meegid.2010.11.002 10.1016/j.meegid.2010.11.002 21111066

[B9] Carvalho DB Almeida CE Rocha CS Gardim S Mendonça VJ Ribeiro AR Alves ZC Ruellas KT Vedoveli A da Rosa JA (2013) A novel association between *Rhodnius neglectus* and the *Livistona australis* palm tree in an urban center foreshadowing the risk of Chagas disease transmission by vectorial invasions in Monte Alto City, São Paulo, Brazil. Acta Tropica 130: 35–38. http://dx.doi.org/10.1016/j.actatropica.2013.10.009 24145156 10.1016/j.actatropica.2013.10.009

[B10] Crossa-Pérez R Hernández M Caraccio M Rose V Valente A Valente V Moreno J Angulo V Sandoval M Roldán J Vargas F Wolff M Panzera F (2002) Chromosomal evolution trends of the genus *Panstrongylus* (Hemiptera, Reduviidae), vectors of Chagas Disease. Infection Genetics and Evolution 2: 47–56. http://dx.doi.org/10.1016/S1567-1348(02)00063-1 10.1016/s1567-1348(02)00063-1 12798000

[B11] Díaz S Panzera F Jaramillo-Ocampo N Pérez R Fernández R Vallejo G Saldaña A Calzada JE Triana O Gómez-Palacio A (2014) Genetic, cytogenetic and morphological trends in the evolution of the *Rhodnius* (Triatominae: Rhodniini) trans-Andean group. PLoS One 9: 87493. http://dx.doi.org/10.1371/journal.pone.0087493 10.1371/journal.pone.0087493 PMC3911991 24498330

[B12] Dujardin JP Costa J Bustamante D Jaramillo N Catala S (2009) Deciphering morphology in Triatominae: the evolutionary signals. Acta Tropica 110: 101–111.https://doi.org/10.1016/j.actatropica.2008.09.026 19026978 10.1016/j.actatropica.2008.09.026

[B13] Dujardin JP Schofield CJ Panzera F (2002) Los vectores de la enfermedad de Chagas. Bruxelles : Académie Royale des Sciences d’Outre-Mer 25(3): 1–189.

[B14] Dujardin J Steinden M Chavez T Machane M Schofeld C (1999) Changes in the Sexual Dimorphism of Triatominae in the Transition From Natural to Artifcial Habitats. Memórias Instituto Oswaldo Cruz 94: 565–569. http://dx.doi.org/10.1590/S0074-02761999000400024 10.1590/s0074-02761999000400024 10446020

[B15] Ferreira RTB Branquinho MR Leite PC (2014) Transmissão oral da doença de Chagas pelo consumo de açaí: um desafio para a Vigilância Sanitária. Vigilância Sanitária em Debate, Rio de Janeiro 2: 4–11. https://doi.org/10.3395/VD.V2I4.358

[B16] Frias-Lasserre D (2010) A new species and karyotype variation in the bordering distribution of *Mepraia spinolai* (Porter) and *Mepraia gajardoi* Frías et al. (Hemiptera: Reduviidae: Triatominae) in chile and its parapatric model of speciation. Neotropical Entomology, Londrina 39(4): 572–583. http://dx.doi.org/10.1590/S1519-566X2010000400017 10.1590/s1519-566x2010000400017 20877994

[B17] Galati EAB (2016) Morfologia e Taxonomia: 2.1, Classificação de Phlebotominae, and 2.2. Morfologia, Terminologia de Adultos e Identificação dos táxons da América. In: Rangel EF, Lainson R (Eds) Flebotomíneos do Brasil, Rio de Janeiro, FIOCRUZ, 2003, 23–51[2.1]; 53–75[2.2].

[B18] Galvão C (2014) Vetores da doença de chagas no Brasil [online]. Sociedade Brasileira de Zoologia, Curitiba, 289 pp https://doi.org/10.7476/9788598203096

[B19] Galvão C Carcavallo R Rocha DS Juberg JA (2003) Checklist of the current valid species of the subfamily Triatominae Jeannel, 1919 (Hemiptera, Reduviidae) and their geographical distribuition, with nomenclatural and taxonomic note. Zootaxa 202: 1–36. http://dx.doi.org/10.11646/zootaxa.202.1.1

[B20] Gómez-Palacio A Jaramillo-Ocampo N Triana-Chávez O Saldaña A Calzada J Pérez R Panzera F (2008) Chromosome variability in the Chagas disease vector *Rhodnius pallescens* (Hemiptera, Reduviidae, Rhodniini). Memórias do Instituto Oswaldo Cruz 103: 160–164. http://dx.doi.org/10.1590/S0074-02762008000200006 18425268 10.1590/s0074-02762008000200006

[B21] Gonçalves TC Teves-Neves SC Santos-Mallet JR Carbajal-de-la-Fuente AL Lopes CM (2013) *Triatoma jatai* sp. nov. in the state of Tocantins, Brazil (Hemiptera: Reduviidae: Triatominae). Memórias do Instituto Oswaldo Cruz 108: 429–37. https://doi.org/10.1590/0074-0276108042013006 23828010 10.1590/0074-0276108042013006 PMC3970630

[B22] Gurgel-Gonçalves R Abad-Franch F Ferreira JB Santana DB Cuba CAR (2008) Is *Rhodnius prolixus* (Triatominae) invading houses in central Brazil? Acta Tropica 107: 90-8. https://doi.org/10.1016/j.actatropica.2008.04.020 18550022 10.1016/j.actatropica.2008.04.020

[B23] Jurberg J Rocha DS Galvão C (2009) *Rhodnius zeledoni* sp. nov. afm de *Rhodnius paraenses* Sherlock, Guitton e Milles, 1977 (Hemíptera, Reduviidae, Triatominae). Biota Neotropica 9: 123–128. https://doi.org/10.1590/S1676-06032009000100014

[B24] Justi AS Galvão C (2017) The Evolutionary Origin of Diversity in Chagas Disease Vectors. Trends Parasitology 33(1): 42–52. http://dx.doi.org/10.1016/j.pt.2016.11.002 10.1016/j.pt.2016.11.002 PMC5518462 27986547

[B25] Lent H Jurberg J (1969) O gênero *Rhodnius* Stål, 1859, com um estudo sobre a genitália das especies (Hemiptera, Reduviidae, Triatominae). Revista Brasileira Biologia 29: 487–560.

[B26] Lent H Wygodzinsky P (1979) Revision of the Triatominae (Hemiptera, Reduviidae), and their significance as vectors of Chagas disease. Bulletin of the American Museum of Natural History 163: 123–520.

[B27] Mendonça VJ Alevi KC Pinotti H Gurgel-Gonçalves R Pita S Guerra AL Panzera F De Araújo RF Azeredo-Oliveira MT Rosa JA (2016) Revalidation of *Triatoma bahiensis* Sherlock & Serafim, 1967 (Hemiptera: Reduviidae) and phylogeny of the *T. brasiliensis* species complex. Zootaxa 4107: 239–254. https://doi.org/10.4269/ajtmh.2009.08-0664 27394816 10.11646/zootaxa.4107.2.6

[B28] Ministério da Saúde, Brazil (2017) Doença de Chagas. http://portalsaude.saude.gov.br/index.php/oministerio/principal/secretarias/svs/doencadechagas

[B29] Nattero J Pita S Calleros L Crocco L Panzera Y Rodríguez CS Panzera F (2016) Morphological and Genetic Differentiation within the Southernmost Vector of Chagas Disease: *Triatoma patagonica* (Hemiptera - Reduviidae). PLoS ONE 11(12): e0168853. http://dx.doi.org/10.1371/journal.pone.0168853 10.1371/journal.pone.0168853 PMC5179239 28005972

[B30] Neiva A Pinto C (1923) Dos hemípteros hematophagos do Norte do Brasil com descrição de duas novas espécies. Brasil Médicina 37: 73–76.

[B31] Obara MT da Rosa JA Ceretti W Urbinatti PR Quintero LO Barata JM Galvão C (2007) A study of the scutellum in eight Chagas disease vector species from genus *Triatoma* (Hemiptera, Reduviidae) using optical and scanning electron microscopy. Memórias do Instituto Oswaldo Cruz 102: 463–468. https://doi.org/10.1590/S0074-02762007005000027 17612766 10.1590/s0074-02762007005000027

[B32] Panzera F Dujardin JP Nicolini P Caraccio MN Rose V Tllez T Bermúdez H Bargues MD Mas-Coma S Connor JH Perez R (2004) Genomic changes of Chagas disease vector, South America. Emerging Infectious Diseases 10: 438–46. doi: 10.3201/eid1003.020812 15109410 10.3201/eid1003.020812 PMC3322799

[B33] Panzera F Fernandis I Ramsey J Ordòñez R Salazar-Schettino PM Cabrera M Monroy MC Bargues MD Mas-Coma S Connor EO Ângulo VM Jaramillo N Cordon-Rosales C Gómez D Perez R (2006) Chromosomal variation and genome size support existence of cryptic species of *Triatoma dimidiata* with different epidemiological importance as Chagas disease vectors. Tropical Medicine & International Health 11: 1092–1103. doi: 10.1111/j.1365-3156.2006.01656.x 16827710 10.1111/j.1365-3156.2006.01656.x

[B34] Panzera F Hornos S Pereira J Cestau R Canale D Diotaiuti L Dujardin JP Perez R (1997) Genetic variability and geographic differentiation among three species of triatomine bugs (Hemiptera-Reduviidae). The American Journal of Tropical Medicine & Hygiene 57: 732–739. https://doi.org/10.4269/ajtmh.1997.57.732 9430537 10.4269/ajtmh.1997.57.732

[B35] Panzera Y Pita S Ferreiro MJ Ferrandis I Lages C Pérez R Silva AE Guerra M Panzera F (2012) High dynamics of rDNA cluster location in kissing bug holocentric chromosomes (Triatominae, Heteroptera). Cytogenetic and Genome Research 138: 56–67. https://doi.org/10.1159/000341888 22907389 10.1159/000341888

[B36] Pita S Panzera F Ferrandis I Galvão C Gómez-Palacio A Panzera Y (2013) Chromosomal divergence and evolutionary inferences in Rhodniini based on the chromosomal location of ribosomal genes. Memorias do Instituto Oswaldo Cruz 108: 376–82. https://doi.org/10.1590/S0074-02762013000300017 10.1590/S0074-02762013000300017 PMC4005576 23778665

[B37] Poinar G Jr (2005) *Triatoma dominicana* sp. n. (Hemiptera: Reduviidae: Triatominae), and *Trypanosoma antiquus* sp. n. (Stercoraria: Trypanosomatidae), the first fossil evidence of a triatomine-trypanosomatid vector association. Vector Borne Zoonotic Diseases 5(1): 72–81. https://doi.org/10.1089/vbz.2005.5.72 15815152 10.1089/vbz.2005.5.72

[B38] Poinar G Jr (2013) *Panstrongylus hispaniolae* sp. n. (Hemiptera: Reduviidae: Triatominae), a new fossil triatomine in Dominican amber, with evidence of gut flagellates. Palaeodiversity 6: 1–8.

[B39] Quintero LO (2002) Avaliação do valor sistemático do processo do I urotergito em machos de onze espécies de importância em saúde pública da subfamília Triatominae (Hemiptera, Reduviidae). PhD thesis, Faculdade de Saúde Pública, Universidade de São Paulo, São Paulo, 85 pp.

[B40] Rodrigues VL Pauliquevis Junior C da Silva RA Wanderley DM Guirardo MM Rodas LA Casanova C Pachioni ML Souza WA Costa AJ Baitelo D Tonietti VL (2014) Colonization of palm trees by *Rhodnius neglectus* and household and invasion in an urban area, Araçatuba, São Paulo State, Brazil. Revista do Instituto de Medicina Tropical de São Paulo 56: 213–218. http://dx.doi.org/10.1590/S0036-46652014000300006 24878999 10.1590/S0036-46652014000300006 PMC4085872

[B41] Rosa JA Mendonça VJ Gardim S Carvalho DB Oliveira J Nascimento JD Pinotti H Pinto MC Cilense M Galvão C Barata JM (2014) Study of the external female genitalia of 14 *Rhodnius* species (Hemiptera, Reduviidae, Triatominae) using scanning electron microscopy. Parasites & Vectors 7: 1–17. https://doi.org/10.1186/1756-3305-7-17 24405517 10.1186/1756-3305-7-17 PMC3896706

[B42] Rosa JA Rocha CS Gardim S Pinto MC Mendonça VJ Ferreira Filho JCR Carvalho EOC Camargo LMA Oliveira J Nascimento JD Cilense M Almeida CE (2012) Description of *Rhodnius montenegrensis* n. sp. (Hemiptera: Reduviidae: Triatominae) from the state of Rondônia, Brazil. Zootaxa 3478: 62–76.

[B43] Rosa JA Mendonça VJ Rocha CS Gardim S Cilense M (2010) Characterization of the external female genitalia of six species of Triatominae (Hemiptera: Reduviidade) by scanning electron microscopy. Memórias do Instituto Oswaldo Cruz 105: 286–292. https://doi.org/10.1590/S0074-02762010000300007 20512241 10.1590/s0074-02762010000300007

[B44] Rosa JA Barata JMS Cilence M Belda Neto FM (1999) Head morphology of 1st and 5th instar nymphs of *Triatoma circummaculata* and *Triatoma rubrovaria* (Hemiptera, Reduviidae). International Journal of Insect Morphology and Embryology 28: 363–375.

[B45] Santos CM Jurberg J Galvão C Rosa JA Júnior WC Barata JM Obara MT (2009) Comparative descriptions of eggs from three species of *Rhodnius* (Hemiptera: Reduviidae: Triatominae). Memórias do Instituto Oswaldo Cruz 104: 1012–1018. https://doi.org/10.1590/S0074-02762009000700013 20027470 10.1590/s0074-02762009000700013

[B46] Schimid M (1980) Chromosoma banding an amphibia IV. Differentiation of GC and AT rich regions in Anura. Chromosoma 77: 83–103. 7371452 10.1007/BF00292043

[B47] Severi-Aguiar GD Lourenco LB Bicudo HE Azeredo-Oliveira MTV (2006) Meiosis aspects and nucleolar activity in *Triatoma vitticeps* (Triatominae, Heteroptera). Genetica 126: 141–151. https://doi.org/10.1007/s10709-005-1443-2 16502091 10.1007/s10709-005-1443-2

[B48] Souza ES Von Atzingen NCB Furtado MB Oliveira J Nascimento JD Vendrami DP Gardim S da Rosa JA (2016) Description of *Rhodnius marabaensis* sp. n. (Hemiptera, Reduviidae, Triatominae) from Pará State, Brazil. ZooKeys 621: 45–62. https://doi.org/10.3897/zookeys.621.9662 10.3897/zookeys.621.9662 PMC5096047 27833419

[B49] Stal C (1859) Monographie der Gattung Conorhinus und Verwandten. Berliner Entomologische Zeitschrift 3: 99–117. http://biostor.org/reference/61560

[B50] Sumner AT (1972) A simple technique for demonstrating centromeric heterochromatin. Experimental Cell Research 75: 304–306. https://doi.org/10.1016/0014-4827(72)90558-7 4117921 10.1016/0014-4827(72)90558-7

